# Evidence for a quadruplex structure in the polymorphic hs1.2 enhancer of the immunoglobulin heavy chain 3’ regulatory regions and its conservation in mammals

**DOI:** 10.1002/bip.22891

**Published:** 2016-08-18

**Authors:** Marco Sette, Pietro D'Addabbo, Geoffrey Kelly, Alessandro Cicconi, Emanuela Micheli, Stefano Cacchione, Anna Poma, Cesare Gargioli, Vincenzo Giambra, Domenico Frezza

**Affiliations:** ^1^ Department of Chemical Sciences and Technology University of Roma “Tor Vergata,” Roma Italy; ^2^ Department of Biology University of Bari “Aldo Moro” Bari Italy; ^3^ MRC Biomedical NMR Centre The Francis Crick Institute, Mill Hill Laboratory London UK; ^4^ Department of Biology and Biotechnology Sapienza University Roma Italy; ^5^ Institute Pasteur‐Fondazione Cenci‐Bolognetti Roma Italy; ^6^ Department of Life, Health and Environmental Sciences University of L'Aquila L'Aquila Italy; ^7^ Department of Biology University of Roma “Tor Vergata,” Roma Italy; ^8^ “Terry Fox” Laboratory BC Cancer Agency Vancouver Canada

**Keywords:** polymorphic enhancer hs1.2, circular dichroism, nuclear magnetic resonance, quadruplex DNA, immunoglobulins

## Abstract

Regulatory regions in the genome can act through a variety of mechanisms that range from the occurrence of histone modifications to the presence of protein‐binding loci for self‐annealing sequences. The final result is often the induction of a conformational change of the DNA double helix, which alters the accessibility of a region to transcription factors and consequently gene expression. A ∼300 kb regulatory region on chromosome 14 at the 3' end (3'RR) of immunoglobulin (Ig) heavy‐chain genes shows very peculiar features, conserved in mammals, including enhancers and transcription factor binding sites. In primates, the 3'RR is present in two copies, both having a central enhancer named hs1.2. We previously demonstrated the association between different hs1.2 alleles and Ig plasma levels in immunopathology. Here, we present the analysis of a putative G‐quadruplex structure (tetraplex) consensus site embedded in a variable number tandem repeat (one to four copies) of hs1.2 that is a distinctive element among the enhancer alleles, and an investigation of its three‐dimensional structure using bioinformatics and spectroscopic approaches. We suggest that both the role of the enhancer and the alternative effect of the hs1.2 alleles may be achieved through their peculiar three‐dimensional‐conformational rearrangement. © 2017 The Authors Biopolymers Published by Wiley Periodicals, Inc. Biopolymers 105: 768–778, 2016.

## INTRODUCTION

A relevant role of noncoding and intergenic regions in the human genome control has been recognized for many years. Emphasis has been given to the function of these regions in genetic regulation.^1^, ^2^, ^3^ This task is achieved either through binding of specific proteins or through inducing specific DNA conformations, which result in the control of entire coding regions.^4^, ^5^

We are interested in understanding how the 3’ regulative region (3'RR) of immunoglobulin heavy chain gene cluster (IgH) works. During B‐cell maturation, the 3'RR plays fundamental control roles for somatic hypermutation, and in class switch through epigenetic changes of chromatin.^6^, ^7^ The 3'RR is present in a single copy in nonprimate mammals, while is duplicated in human on chromosome 14, and in most primate species on related syntenic regions.^8^ Despite synteny among 3'RRs in different mammals, these regions can harbor three enhancers (hs1.2, hs3, and hs4) as in humans, or four enhancers, one of the original three being duplicated, as in rodents, in which also three insulators (hs5, hs6, and hs7) are present in a lineage‐specific sequence.^9^ In all species, hs1.2 maps to the central conserved core of the 3'RRs. In the genomes harboring the duplication, binding sites for specific transcription factors like NF‐kB and Sp1, are conserved in both the paralogous copies of the hs1.2 enhancer.^8^ These sites and their peptidic interactors are thought to play a crucial role both in the isotipic switch and in correct Ig transcription at different stages of B‐cell development.^6^, ^7^, ^10^, ^11^, ^12^, ^13^ In agreement with these findings, we demonstrated that human polymorphisms of hs1.2 enhancer in 3'RR1 are associated with the humoral‐response control of Ig‐circulating levels and with autoimmune diseases.^14^, ^15^, ^16^ Moreover, an imbalance of allele frequency is observed in celiac disease, psoriasis, systemic sclerosis, rheumatoid arthritis, and lupus erythematosus patients when compared with healthy controls.^16^, ^17^, ^18^, ^19^, ^20^

Despite their inferred role, we still lack knowledge of the structural and functional details of 3'RRs. In vivo experiments, like fluorescence in situ hybridization and chromosome conformation capture, confirmed a large conformational rearrangement of the DNA, although these methods did not provide specific structural information.^4^, ^21^ A first insight into the three‐dimensional (3D) structure of the 3'RR was given by previous in silico studies that showed the presence of an inverted duplication. As a palindromic sequence, the inverted duplication may give rise to a hairpin‐loop shape, in which the hs1.2 enhancer is located at the apical loop.^8^, ^22^, ^23^ The inverted duplication can be recognized in all mammals studied so far, although it does not show similarity among species belonging to different orders, supporting the hypothesis of a function linked to specific 3D structures.^8^, ^24^ The hypothesized 3D structure locates the hs1.2 enhancer in the middle of a loop where the DNA is supposed to assume single‐stranded conformation. The human version of this enhancer has allelic variants with an internal Variable Number Tandem Repeat (VNTR), consisting of a 40 nucleotides element (40‐mer) repeated from one to four times. Studies aimed at defining a list of the human genetic variations using whole genome and multigenome approaches seem to fail to highlight the peculiarity of these VNTR until recently.^25^ The 40‐mer contains a binding site for NF‐kB and SP1, as demonstrated by electrophoretic mobility shift assays.^14^, ^19^, ^26^ This finding suggests a relevant biological role of the enhancer.

In this work, we analyze in detail a 17‐nucleotides DNA sequence (17‐mer) spanning a portion of the enhancer hs1.2 that is predicted to form a quadruplex. The 17‐mer maps just upstream to the NF‐kB and SP1 binding sites and, as a potential quadruplex, can have a relevant regulative role on adjacent transcription factors. Genome wide analyses indeed reported both the proximity of putative quadruplex sequence to transcription factor consensus sites^27^ and the association between polymorphisms on quadruplex locus and gene expression.^28^, ^29^ Using our strategy, that combines sequence and spectroscopic analysis, we confirmed the ability of this DNA sequence to form in vitro a quadruplex structure and showed that this feature of the enhancer is preserved from evolutionary divergence. This short sequence is thus a strong candidate to constitute a further fundamental component that finely tunes the hs1.2 activity with protein binding properties similar to many regulatory regions as reported by genomic studies.^30^

## RESULTS

### Analysis of 3'RRs and hs1.2 Main Features, and Schematization of Human Structures

**Figure 1:**
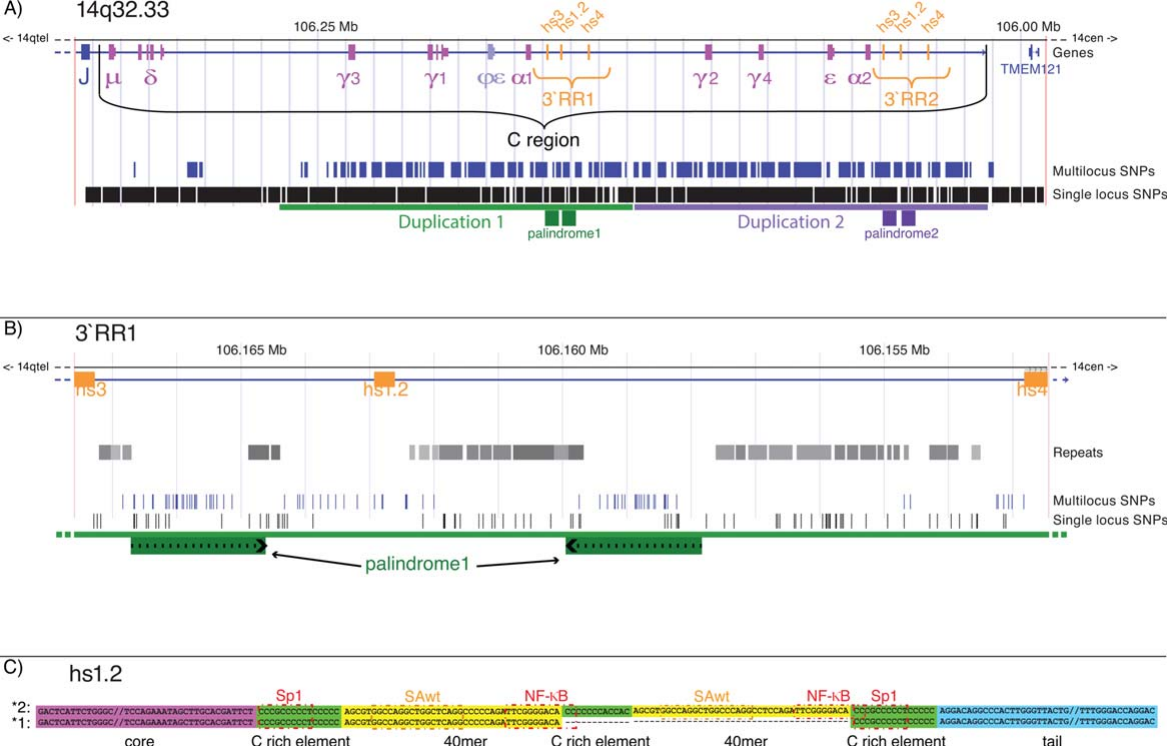
Human 3'RRs and hs1.2‐sequence details. (A) Graphic details of the human Ig constant region (C region), harboring the two copies of the 3’ Regulatory Region (3'RR1 and 3'RR2) as part of extended in‐tandem duplication involving most of the C region. In the two regulatory regions, the two blocks of both palindrome1 (enlarged in section B) and palindrome2 are the inverted repeated branches of the palindromes cited in the text. Internal to both the 3'RRs are the three enhancers, respectively hs3, hs1.2, and hs4, in orange. The blue arrow shows the transcription direction of the Ig genes. In the two bottom lines, the positions of SNPs from the dbSNP build 137. (B) Portion of the human 3'RR1 in higher detail. In green the two branches of the conserved palindromic sequence, embedding a region including hs1.2 with the potential to fold in a big loop of a hairpin structure. (C) Sequences of hs1.2 allele *1 and *2, illustrating VNTR structure, transcription factor consensus sites, and quadruplex putative sites. The main difference between the two isoform is the presence of a different number of the 40mer and C rich elements.

Figure 1 shows a scheme of the human regulative regions (3'RR1 and 3'RR2), located downstream of the IgH α‐gene, ranging from a broad view (Figures 1A and 1B) to details of the nucleotide sequence of the main untranslated feature of the regions, i.e. the enhancer hs1.2 (Figure 1C).

We first considered dbSNP build 137^31^ to investigate the possibility of distinguishing the two paralogous copies of the 3'RR in human NGS data which would give us the chance to discern the two loci in 1K Genome Project data. We concluded that it is impossible to distinguish between the two copies because of the high level of similarity between the 3'RR1 and 3'RR2. Figure 1B, indeed, shows SNPs in the enhancer regions, distinguishing between those which map to a single genomic region from those mapping in multiple genomic loci (multilocus), as reported in dbSNP build 137. Our preliminary re‐sequencing trial (data not shown) of 3'RR2 indicate that some of the SNPs labeled as “single” are probably multilocus or misassigned, beside the already conspicuous number of multilocus SNPs indicated in the figure. These findings suggest uncertainty about the SNP locations making it impossible to distinguish if both, either or neither of the 3’ RRs is conserved within the human species. A possible explanation is that the alignment of short reads from massive sequencing to a reference genome and the consequent identification of polymorphic loci are error‐prone in regions that are duplicated such as the 3'RR, and especially close to the hs1.2 enhancers, because of the high similarity level between its evolutionary‐recent paralogous copies.

The hs1.2 enhancer maps to the conserved center of each 3'RR copy (Figure 1B). The core and tail fragments of the enhancer are the more and the less conserved regions of this functional sequence, respectively, when mammal DNAs are compared with each other.^8^ Between core and tail of hs1.2 there is a region that, in Catarrhini's variant, showed multiple alleles due to an embedded VNTR of 40 nucleotides, repeated in human version of the enhancer from one to four times. This 40‐mer contains binding sites for transcription factors, among which are NF‐kB and SP1 (Figure 1C), known as mainly involved in cellular responses to stimuli such as bacterial or viral antigens and to a growing list of cellular stresses.^32^ Moreover, the 40‐mer contains a putative G‐quadruplex site. The multiplication of the 40‐mer in the various alleles is thus responsible for the proliferation of sequence of putative transcription‐factor‐binding and G‐quadruplex sites within the enhancer.

### Conservation Among Mammals of G‐Quadruplex Predicted in hs1.2

Bioinformatics evidence shows that putative quadruplex regions are often found close to transcription factor binding sites.^30^, ^33^ We thus scanned the 3'RRs in search of putative quadruplex sites and we found evidence for it within hs3, hs1.2, and hs4, the 3'RRs enhancers that are rich in transcription factor binding sites. We focused our attention on the G‐quadruplex inside hs1.2 because of its multiple copies, a feature observed in most primates.^8^ In fact, we noted that the four pairs of guanines putatively forming the quadruplex, are in the VNTR element (Figure 1C), and downstream from a core sequence highly conserved in mammals.8 Figure 1C reports the 17‐mer (GGCCAGGCTGGCCCAGG labeled as SAwt) including the four GG‐pairs within the hs1.2 enhancer that was predicted to form the G‐quadruplex. By comparing sequences available in the GenBank database, we found that the SAwt sequence within the hs1.2 is highly conserved among mammals (Table 1), with a strong constraint on the guanine pairs necessary for the formation of the quadruplex structure. Some species show the contemporary loss of one of the “canonical” G‐pair, and gain of a new G‐pair, so the potential to form quadruplex in the 40‐mer is unaltered. This finding confirms a restriction to the variability of these four pairs of nucleotides and, consequently, the putative functional role of this element in the enhancer sequence. It should be noted that mouse is the only species that has lost two of the G‐pairs in the 40‐mer, so the quadruplex site might actually not be present in mice. The rodents, however, evolved a peculiar regulatory mechanism involving three lineage‐specific insulators^9^ that could complement the quadruplex absence.

**Table 1:** Multiple Alignments of Mammal Sequences

Species—Sequence Reference	1	2	3	4	5	6	7	8	9	10	11	12	13	14	15	16	17	18	19	20	21	22	23	24	25	26	27	28	29	30	31	32	33	34	35	36	37	38	39	40	41	42	43	44	45	46	47	48	49	50	51	52	53	54	55	56	57	58	59	60	61	62
Man—chr14:106,041,686–106,041,724	A	G	T	G	‐	T	G	G	C	C	A	G	G	C	T	G	G	C	C	C	A	G	G	C	C	T	C	C	A	G	‐	A	T	T	C	G	G	G	G	A	C	A	c	c	c	c	c	c	G	c	c	c	c	c	T	c	c	c	c	c	A	G
Orangutan–741916370	A	G	T	G	‐	T	G	G	C	C	A	G	G	C	T	G	G	C	C	C	A	G	G	C	C	T	C	C	A	G	‐	A	T	T	C	G	G	G	G	A	C	A	‐	‐	‐	c	c	c	A	c	c	c	c	c	T	c	c	c	c	‐	T	G
Chimpanzee–232367427	A	G	C	G	‐	T	G	G	C	C	A	G	G	C	T	G	G	C	C	C	A	G	G	C	C	T	C	C	A	G	‐	A	T	T	C	G	G	G	G	A	C	A	‐	‐	‐	c	c	c	G	c	c	c	c	c	T	c	c	c	c	c	c	G
Gorilla—2028842221 & 1672300632	A	G	C	G	‐	T	G	G	C	C	A	G	G	C	T	G	G	C	C	C	A	G	G	C	C	T	C	C	A	G	‐	A	T	T	C	G	G	G	G	A	C	A	‐	‐	‐	c	c	c	G	c	c	c	c	c	T	c	c	c	c	c	c	G
Baboon—1988915424	A	G	C	G	‐	T	G	G	C	C	A	G	G	C	T	G	G	C	C	C	A	G	G	C	C	T	C	C	A	G	‐	A	T	T	C	A	G	G	G	A	C	A	‐	‐	‐	c	c	T	G	c	c	c	c	c	T	c	c	c	c	c	c	G
Gibbon—203887404	A	G	T	G	‐	T	G	G	C	C	A	G	G	C	T	G	G	C	T	C	A	G	G	C	C	T	C	C	A	G	‐	T	T	T	T	G	G	G	G	A	C	A	‐	‐	‐	c	c	c	G	c	c	c	c	c	T	A	c	c	c	c	c	G
Macaque—657888373	A	G	C	A	‐	T	G	G	C	C	A	G	G	C	T	G	G	C	C	C	C	G	G	C	C	T	C	C	A	G	‐	A	T	T	C	G	G	G	G	A	C	A	‐	‐	‐	c	c	c	G	c	c	c	c	c	T	c	c	c	c	c	c	G
Galago—2001951424	A	G	C	A	‐	T	G	T	C	C	A	G	G	C	T	G	G	C	C	C	A	G	G	C	C	T	T	A	G	G	‐	A	T	T	G	G	G	G	G	A	C	‐	‐	‐	‐	c	c	T	C	T	c	c	c	c	T	A	c	c	c	c	c	G
Marmoset—1183332846	G	G	T	G	‐	T	G	G	C	C	A	G	G	C	C	G	G	C	C	C	A	G	G	C	C	T	C	C	A	G	‐	A	T	T	G	G	G	G	G	G	‐	‐	c	c	A	c	c	A	G	c	c	c	c	c	T	‐	‐	‐	‐	‐	c	G
Dolphin—1421106174	G	G	T	G	‐	T	G	G	‐	C	G	G	G	T	T	G	G	C	C	T	A	G	G	C	C	T	C	G	G	G	G	‐	A	T	C	G	G	G	G	G	G	C	c	G	A	G	c	c	G	c	c	G	G	G	‐	‐	‐	c	c	c	c	C
Alpaca—KB633178	A	G	C	A	‐	T	G	G	C	C	A	G	G	C	T	G	G	C	C	‐	A	G	G	C	C	T	C	A	G	G	‐	A	G	T	G	G	G	G	G	‐	‐	‐	c	c	c	c	T	G	A	G	c	c	c	c	T	c	c	c	c	c	c	G
Dog—1301781749	A	C	T	G	C	A	G	G	C	C	A	G	G	T	G	G	G	C	C	C	A	G	G	C	C	T	C	G	G	G	‐	C	T	C	A	G	G	G	C	C	C	T	c	T	G	c	c	c	A	A	c	c	c	c	‐	c	c	G	c	c	G	G
Horse—1440906082	A	G	C	A	‐	C	G	G	C	C	T	G	G	C	T	G	G	‐	C	C	A	G	G	C	C	T	C	A	G	G	C	C	T	T	G	G	G	G	G	A	‐	‐	‐	‐	‐	c	c	c	T	T	c	c	c	c	T	c	c	c	c	c	c	G
Pig—AAKN02042605	T	G	G	T	‐	T	G	G	C	C	A	G	G	‐	T	G	G	C	C	C	A	G	G	C	C	T	C	‐	A	G	G	A	C	T	T	G	G	A	G	A	C	‐	‐	‐	c	c	c	T	G	c	c	c	A	c	T	‐	‐	‐	c	c	c	G
Panda—ACTA01092430	A	G	C	A	‐	C	G	G	C	C	A	G	G	T	T	A	G	C	C	C	C	G	G	C	C	T	C	‐	A	G	G	G	T	T	C	G	G	G	G	‐	C	A	G	c	c	c	T	c	C	A	c	c	c	c	T	c	c	c	‐	‐	c	G
Cat—chrB3:148,031,190–148,031,242	A	G	C	A	‐	T	G	G	C	C	A	G	G	T	T	G	G	C	C	T	A	G	G	C	C	T	‐	T	G	G	G	‐	T	T	C	G	G	G	G	G	‐	A	c	c	c	T	c	c	A	‐	c	c	c	c	T	G	c	c	‐	‐	‐	‐
Cattle—632080601	A	G	T	‐	‐	‐	‐	G	C	C	A	G	G	C	T	G	G	C	C	‐	T	G	G	A	G	C	T	C	A	G	G	A	T	T	C	G	G	G	G	G	‐	A	c	c	c	T	G	C	A	c	c	c	c	c	T	G	c	c	c	c	c	G
Deer mouse—2189185153	A	T	G	C	‐	T	G	G	C	C	A	G	G	T	T	G	G	C	C	T	A	G	G	C	T	T	T	G	G	G	G	G	‐	T	G	G	G	G	G	A	C	‐	‐	‐	‐	‐	‐	‐	‐	‐	‐	c	c	c	T	c	c	c	‐	‐	c	G
Mouse—chr12:113,244,756–113,244,806	A	A	C	C	‐	T	G	G	C	C	A	G	G	C	T	G	A	‐	C	C	A	G	‐	C	C	T	T	G	G	G	G	A	T	‐	G	G	G	G	G	A	C	T	‐	‐	c	c	c	A	G	c	‐	‐	‐	‐	‐	‐	‐	‐	A	T	c	G

Nucleotides from 1 to 42 correspond to the human 40‐mer; the short C‐rich locus that completes the repeated element of hs1.2 is numbered 43 to 62.

### Native Electrophoresis of the Putative Quadruplex Regions

In order to get insights on the behavior in solution of the SAwt sequence, we performed native polyacrylamide gel electrophoresis (PAGE). We decided to buffer the solutions with potassium as a counter‐ion to exploit the known tendency of this ion to stabilize quadruplex formation through its ability to bind between the guanine quartets.^34^

**Figure 2:**
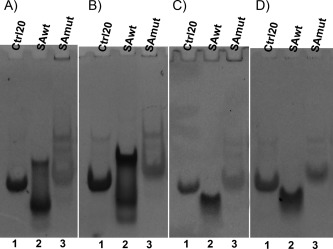
PAGE analysis of the quadruplex region. Native 15% PAGE of SAwt, SAmut and Ctrl20 oligonucleotides in different pH and salt conditions. A) pH 6.5, no KCl; B) pH 6.5, 100 m*M* KCl; C) pH 4.5, no KCl; D) pH 4.5, 100 m*M* KCl. In each panel, lane 1 corresponds to a 20‐nucleotide oligonucleotide unable to form G‐quadruplex, used as a control, while lanes 2 and 3 correspond to SAwt and SAmut, respectively.

Figures 2A and 2B show the PAGE of SAwt at 5 and 100 m*M* potassium ion concentration and pH 6.5.

Under both conditions, SAwt shows a smearing of bands of several molecular weights.

Two more prominent bands, migrating slower and faster than the unfolded 20‐mer oligonucleotide used as a control, are observed.

Thus, the data indicate the presence of multiple species in solution.

A possible explanation is the presence of multiple cytosines in the SAwt sequence and then the possibility of impaired duplex formation. An example is reported in Scheme 1.

In order to exclude the formation of impaired duplex and to verify that the sequence studied can form a quadruplex structure, two different strategies were undertaken:

- Analysis of the SAwt sequence at low pH, where the formation of double strand is hampered;
- Study the behavior of a similar sequence, in which all the cytosines are replaced by thymines, maintaining the correct spacing between guanines nucleotides. Such a sequence will be defined as SAmut (GGTTTGGTTGGTTTTGG).

Here below, both approaches are evaluated in parallel for all techniques used.

Concerning the natural sequence, SAwt, at pH 4.5 (Figures 2C and 2D) no heterogeneity is observed and a single band is present, migrating faster than the control. This band could be attributed to a quadruplex structure since in the presence of saturating amount of potassium, which is known to induce the formation of quadruplex structures, the intensity of this band increases, suggesting a stabilization of the structure.

As far as the SAmut fragment is concerned, the band observed at pH 6.5 did not give a clear indication. However, the observation that at a pH as low as 4.5, a single band of low mobility is observed suggests the formation of a single structure, different from the one observed for the SAwt fragment since they have different mobility.

### CD Analysis of the Putative Quadruplex Regions

**Figure 3:**
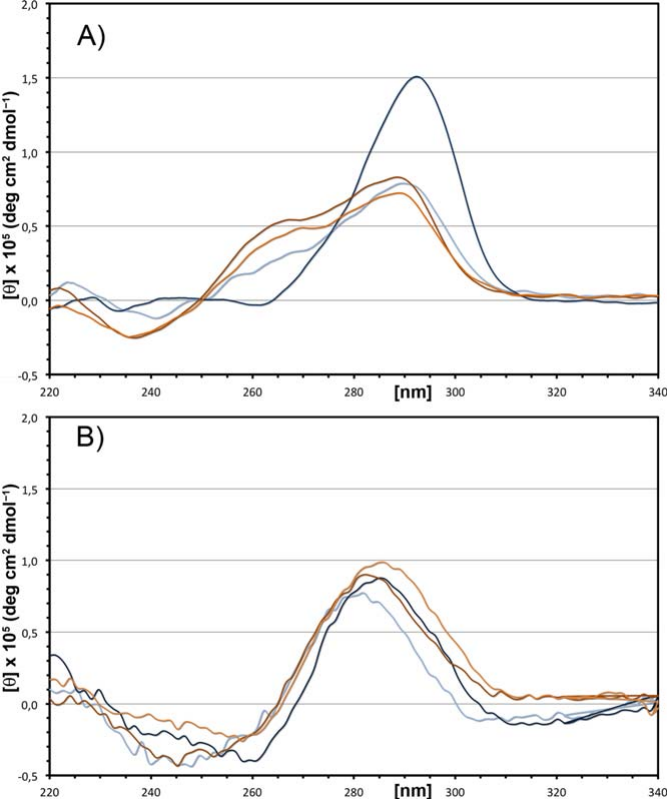
CD spectra of the quadruplex region. CD spectra of (A) the wild‐type 17‐mer DNA oligonucleotide used in this study (SAwt) and (B) the mutated 17‐mer DNA oligonucleotide in which all nonguanine nucleotides are substituted with thymines (SAmut). The spectra are recorded at different pH and potassium concentration. The spectra at pH 6.5 are the brown (5 m*M* potassium phosphate buffer) and the orange line (5 m*M* potassium phosphate buffer, 100 m*M* KCl). The spectra at pH 4.5 are the light blue (5 m*M* potassium acetate buffer) and the dark blue line (5 m*M* potassium acetate buffer, 100 m*M* KCl). In (A) the two positive peaks at 260 nm and 290 nm are indicative of G‐C double strand formation while the small negative and positive peaks at 260 nm and 290 nm are indicative of unimolecular quadruplex. In (B) the negative and positive peaks at 260 nm and 290 nm are indicative of quadruplex formation at pH 4.5.

Figure 3A shows the CD spectra of the native SAwt oligonucleotide at pH 6.5 or 4.5.

At pH 6.5 the spectrum shows two positive peaks around 270 and 285 nm and a negative peak at 240 nm. Addition of saturating amounts of potassium had no effect on the spectrum. It seems difficult to give an unambiguous interpretation of these spectra, but some features resemble the poly(dGdC)‐poly(dGdC) and poly(dG)‐poly(dC) previously observed with different oligonucleotide DNA sequences.^35^, ^36^ At pH 4.5, where the formation of double strands is hampered, the spectrum of SAwt shows a negative peak around 260 nm and strong positive peak at 290 nm that clearly indicate the presence of a quadruplex structure.^37^, ^38^ Addition of saturating amounts of potassium has no further effects on the spectrum.

The previous data show that the oligonucleotide at pH 6.5 might form pairings typical of B‐DNA, probably deriving from the formation of impaired intermolecular pairings as suggested by the previous electrophoresis data.

This undesirable effect clearly occurs since hundreds of billions of molecules are present in vitro but cannot occur in vivo where only two strands are present. Thus, we synthesized a different oligonucleotide, SAmut (GGTTTGGTTGGTTTTGG), in which all cytosines and adenines have been substituted by thymines but maintaining the right position of guanines. The CD spectrum of this oligonucleotide is shown in Figure 3B. Both at pH 6.5 and at pH 4.5, in the absence or in the presence of saturating amounts of potassium, the spectra show a positive peak around 290 nm indicating the presence of preformed quadruplex structures.

### NMR Analysis of the Putative Quadruplex Regions

**Figure 4:**
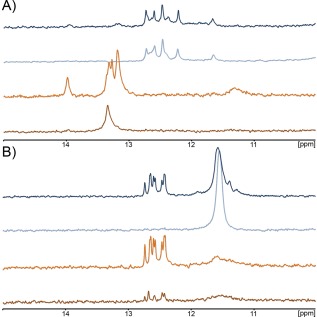
NMR spectra of the quadruplex region. ^1^H NMR spectra of (A) SAwt and (B) SAmut recorded at different pH and potassium concentrations. Brown spectra refers to pH 6.5, 5 m*M* potassium phosphate buffer; orange spectra refers to pH 6.5, 5 m*M* potassium phosphate buffer, 100 m*M* KCl; light blue spectra refers to pH 4.5, 5 m*M* potassium acetate buffer; dark blue spectra refers to pH 4.5, 5 m*M* potassium acetate buffer, 100 m*M* KCl. Peaks from 13.0 to 14.0 ppm are indicative of double strand formation; narrow peaks between 11.5 and 13.0 ppm are indicative of quadruplex formation. The broad peak around 11.5 ppm belongs to unstructured DNA.

Figure 4A shows the NMR spectra collected at 27°C for SAwt at 200 μ*M* concentration and different pH values in the absence or in the presence of 100 m*M* KCl. At pH 6.5, the chemical shifts observed for the imino protons are in 13.00 to 14.00 ppm range, indicative of the formation of double‐stranded DNA and/or G•C•G•C tetrads;^39^ there is no evidence of quadruplex formation, characterized by a different chemical shift range for imino protons. Since the isolated SAwt oligonucleotide is not autocomplementary but contains several cytosines, we attributed this secondary structure to the formation of impaired alignments (see Scheme 1). This is in agreement with the results obtained by CD and electrophoresis analysis. The number and intensities of peaks in the 13.00 to 14.00 ppm range increase after the addition of 100 m*M* potassium. Similar spectra were collected at lower concentration of DNA (10 μ*M*, data not shown) to shift the equilibrium toward the single strand in the absence of potassium and possibly toward quadruplex in the presence of potassium but the results were the same, indicating that impaired double strands are very stable. The sample in the presence of a saturating amount of potassium was then kept at 95°C for 10 min and then slowly allowed to anneal but the final secondary structure observed still suggests the presence of double‐stranded DNA (data not shown). At lower pH, where the formation of double strands is hampered, the SAwt oligonucleotide shows imino protons peaks in the range 12.00 to 13.0 ppm that are clearly indicative of quadruplex secondary structure, even in the presence of low amounts of potassium. Since potassium is present in the potassium acetate buffer, the formation of quadruplex structure appears already at minimal amounts of potassium. This is in agreement with the CD data that suggest a quadruplex structure under these conditions (Figure 3). Increasing the potassium concentration induces small but detectable changes in the NMR spectrum with small shift of some resonances and different relative intensity of some signals. This could indicate a slight rearrangement of the structure that could well explain the differences observed in the CD spectra. Furthermore, low intensity signals are observed in the same position of the signals observed at pH 6.5. Such signals could indicate a minor form in equilibrium with the quadruplex.

To verify that the double‐stranded structure observed at pH 6.5 arises from impaired GC alignment and “Watson‐Crick” bonds we also collected NMR spectra of SAmut, the mutant oligonucleotide in which all adenines and cytosines were replaced by thymines. The position of guanines is maintained in this oligonucleotide but impaired alignments cannot be formed. The spectra of this oligonucleotide at different pH values are reported in Figure 4B and show that the imino protons peaks are in the range 11.5 to 12.5 ppm in the presence of potassium both at neutral and at acidic conditions. This finding indicates the absence of B‐DNA formation and the formation of a typical quadruplex DNA in all conditions. The broad peak around 11.5 ppm is indicative of residual, unfolded, DNA.

These results clearly indicate the formation of quadruplex structures for both DNA oligonucleotides.

### Thermal Denaturation of the Putative Quadruplex Region

**Figure 5:**
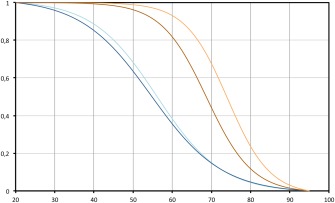
Melting analysis of the quadruplex region. CD Melting curves of SAwt collected at different pH and different potassium concentrations. Brown, pH 6.5, 5 m*M* potassium phosphate buffer; orange, pH 6.5, 5 m*M* potassium phosphate buffer, 100 m*M* KCl; light blue, pH 4.5, 5 m*M* potassium acetate buffer; dark blue, pH 4.5, 5 m*M* potassium acetate buffer, 100 m*M* KCl.

To gain further insight we analyzed the melting profiles of the SAwt fragment present in the human genome. Figure 5 shows the melting curves of SAwt. At pH 4.5 the calculated *T*_m_ at 5 m*M* and 100 m*M* potassium are about 55.5°C and 54.8°C, respectively. These values are compatible with the formation of a unimolecular quadruplex as suggested by the previous data. At pH 6.5 the *T*_m_ in the presence of 5 and 100 m*M* potassium are 68.5°C and 73.8°C, respectively. These values are unusually high for a 17‐mer and may reflect the formation of multistranded DNA structures.

### SAwt Oligonucleotide Sequence is Conserved Among Primates and Mammalians

We identified six loci in the human genome showing 100% identity with the SAwt sequence of hs1.2 enhancers in the 3′RRs (Table 2). One of the six loci maps in a large gene‐desert region with unidentified function, a second one is within the translated sequence of DCAF12L1 (a member of the WD repeat protein family), and the four others map in transcribed regulatory regions, just like the two hs1.2 copies (Table 2). However, when alignment to primate or mammal genome of each of the SAwt‐similar loci is considered, some interesting indications can be found. In nonhuman primates, all six sequences show some variation with respect to the SAwt sequence that alter the four GG pairs needed for the quadruplex formation, suggesting relaxed selection in these species at those loci. By contrast, when the 3'RR1 and 3'RR2 loci are considered, the GG pairs are conserved in all primates and in many mammal genomes (Tables 1 and II), suggesting a strong negative selection against mutations at these positions and in turn a possible conserved functional role of this fragment of the enhancer.

**Table 2:** Human Genomic Regions Identical to the SAwt Oligonucleotide, and Conservation of These Putative Quadruplex Loci in Mammal Genomes

						GG‐pairs conservation							
						Primate				Others Mammals			
chr	Start	End	Strand	Involved Genes	Chromatin State Segmentation	G1	G2	G3	G4	G1	G2	G3	G4
2	129064998	129065014	+	HS6ST1 (intron)	Strong enhancer in epidermal keratinocytes (NHEK)	Y	Y	Y	N	—	—	—	—
5	1642717	1642733	+	—	Weak/poised enhancer in embryonic stem cells (H1‐hESC)	Y	Y	N	N	—	—	—	—
6	155541590	155541606	+	TIAM2 (intron)	Weak/poised enhancer in three cell‐lines (HUVEC, HMEC, HSMM)	N	Y	Y	N	N	N	N	Y
12	124571796	124571812	+	ZNF664‐FAM101A (intron)	Insulator in lung fibroblasts (NHLF)	**Y**	**Y**	**Y**	**Y**	N	Y	N	Y
14	106041694	106041710	—	—	hs1.2 in 3'RR2: strong enhancer in B‐lymphocyte, lymphoblastoid (GM12878)	**Y**	**Y**	**Y**	**Y**	Y	Y	N	Y
14	106162771	106162787	+	—	hs1.2 in 3'RR1: strong enhancer in B‐lymphocyte, lymphoblastoid (GM12878)	**Y**	**Y**	**Y**	**Y**	—	—	—	—
15	82202427	82202443	—	—	—	Y	Y	Y	N	Y	N	Y	N
X	125686156	125686172	+	DCAF12L1 (exon)	Active promoter in umbilical vein endothelial cells (HUVEC)	N	N	Y	N	Y	Y	N	Y

The SAwt sequence was searched in human genome to find similar putative‐quadruplex loci, and then each locus was searched in mammals to detect corresponding orthologous loci. Five out of six human sites not belonging to the chr14 have the four guanines pairs (G1, G2, G3, and G4) not conserved in primates. The finding suggests higher positive pressure acting on the chr14 and chr12 sequences. Data in the Chromatin State Segmentation column are from ENCODE ChIP‐seq experiments.^40^

## DISCUSSION

Different DNA conformations modify the availability of consensus sequences for the binding of specific proteins and 3D structures in the binding region which can finely tune the binding process.^5^, ^27^, ^30^, ^41^, ^42^, ^43^ In many cases, the DNA molecule must be in an open and accessible state to allow the binding of specific proteins and the 3D structures cooperate to define the actual condition of the embedded consensus sites. This process has been called chromatin remodeling and requires the activity of proteins necessary to maintain the chromatin in an accessible state for nuclear factors like NF‐kB.^41^ Specific chromatin modifications and configurations are in fact necessary for NF‐kB proteins to bind cognate kB motifs.^28^ Thus, the presence of kB sites appears to be a requirement but is not sufficient for gene induction.^40^ The effectiveness of the consensus site is influenced by the genomic and nuclear environment in which it is embedded.^42^

We previously observed the possibility of a 3D palindromic structure that could modulate the role of the 3'RR by formation of a hairpin structure, in which the hs1.2 enhancer locates at the middle of the apical loop.^8^ This site was probably also tandem‐repeated in the Catarrhini‐parvorder ancestor, in which also the VNTR of hs1.2 originated.^8^ These remarks suggest that the current version of the enhancer may have evolved from an ancestral sequence including the core and a single copy of the quadruplex site.

In this article, we show that a quadruplex structure might form inside the hs1.2 enhancers in the 3'RRs (Figures 2 and 3) and we speculate a possible functional role for it in exposing DNA in order to modulate the binding of transcription factors. We show that the hs1.2 short oligonucleotide required for the quadruplex structure formation is highly conserved in primates and mammals, unlike other identical sequences in the human genome, suggesting that strong negative selection is acting on this region and corroborating the hypothesis of a functional role of the quadruplex 3D structures.

The formation of a quadruplex structure indicates the presence of a newly identified functional region within the hs1.2 enhancer. Quadruplex formation in Igs had been speculated many years ago,^44^ and the present work gives some experimental evidence of such structure. This finding allows the formulation of hypotheses on the function of this polymorphic enhancer suggesting a possible modulating effect in the equilibrium among the alternative 3D forms that can modulate the binding of NF‐kB and SP1 nuclear factor. The predicted quadruplex sequence in fact lies just upstream of the NF‐kB transcription factor consensus site (Figure 1C). In this context it must be noted that both NF‐kB and Sp1 bind to double‐stranded DNA. Thus, a possible role of the quadruplex structure may be to shift the equilibrium of the 3'RR region towards the hairpin structure, stabilizing this alternative conformation of the DNA. The final effect of this conformational change is the inhibition of transcription factors binding since the DNA is single‐stranded. An alternative possibility is the formation of a local 3D structure, the quadruplex, along the double‐stranded helix, which creates a steric hindrance to the binding of transcription factors to their consensus sites.

These results support our previous findings on physiologic and pathologic phenotypes that clarified the modulatory function of the hs1.2 alleles in Ig production. Taken together, these studies reveal the relevant role of the 3D structures of DNA for regulation of Igh expression and B cell activity.

## MATERIALS AND METHODS

### In Silico Analyses of hs1.2 Sequence

We searched for copies of the hs1.2 enhancer in three Genbank databases (i.e. genome, htgs, and wgs) by the NCBI Blast algorithm (http://blast.ncbi.nlm.nih.gov/).^50^ The Genomic Evolutionary Rate Profiling (GERP) score, Single Nucleotide Polymorphisms (SNPs), repeat, and 3'RRs features are analyzed, identified and/or graphically visualized using the UCSC Genome Browser^46^ (http://genome.ucsc.edu/cgi-bin/hgGateway). The G‐quadruplex structure consensus sites were identified by Quadbase^47^ (http://quadbase.igib.res.in) and QGRS Mapper^48^ (http://bioinformatics.ramapo.edu/QGRS).

### Spectroscopic Analysis of the DNA Sequence

For the in vitro analysis we used two oligonucleotides derived from the polymorphic region of hs1.2 enhancer in the human 3'RRs (Figure 1A; detailed view of the 3'RR1 in Figure 1B): a sequence of 17 nucleotides (17‐mer, SAwt: GGCCAGGCTGGCCCAGG) selected as a G‐quadruplex putative consensus sequence from in silico analysis (Figure 1C); the second oligonucleotide was designed to replace all the C and A in the SAwt with T (SAmut: GGTTTGGTTGGTTTTGG). Both oligonucleotides were synthesized by Eurofins (Milano, Italy), purified by HPLC and desalted. Samples were denatured at 95°C for 10 min and then slowly cooled before use. Circular dichroism (CD) and nuclear magnetic resonance (NMR) spectra at pH 6.5 were obtained by dissolving the sample in 5 mm potassium phosphate buffer, while the spectra at pH 4.5 were obtained by dissolving the sample in 5 m*M* potassium acetate buffer. KCl was added from 3*M* stock solutions and spectra were collected soon after the addition of potassium. CD spectra were collected on a Jasco J600 Spectrometer (Jasco Inc., Easton, MD) using a 0.1 cm path cell at 300 K. Data were obtained from 200 to 340 nm at 0.2 nm interval, 20 nm/min speed, and averaging over four scans. Samples contained 20 μ*M* of DNA. Data are reported as mean residue ellipticity. NMR spectra were collected using concentrations ranging from 10 to 200 μ*M* at 400 or 600 MHz on Bruker or Varian spectrometers, respectively. A 10% D_2_O was used for lock and water was suppressed by using a watergate pulse sequence^49^ that gives a maximum excitation over the imino proton region. The spectra were collected at different temperatures ranging from 2 to 27°C and analysis was done using the MNova software (Mestrelab Research, Spain).

### Polyacrylamide Gel Electrophoresis (PAGE)

PAGE analysis has been carried out as previously described.^50^ The samples (5 µ*M* oligonucleotides in 5 m*M* potassium acetate buffer, pH 4.5 or 6.5) were heated to 95°C for 10 min, and then annealed by slowly cooling to room temperature with or without 100 m*M* KCl. After incubation at 4°C for at least 1 h, the samples were run on 18% native PAGE (19:1 acrylamide:bisacrylamide ratio) with TAE as electrophoretic running buffer, containing the same salt concentration of the run samples. Each run was performed at room temperature for about 1 h and 30 min at 130 V. The gels were stained with SYBR Safe DNA gel stain (Invitrogen).

### CD Melting Analysis

CD spectra were recorded using a JASCO J‐715 spectropolarimeter equipped with a Peltier temperature controller, using a quartz cell of 1.0 cm optical path length, in the range 20 to 95°C, increasing the temperature with a 1°C increasing step per minute. The CD melting profiles were obtained plotting the molar ellipticity corresponding to the maximum at about 290 nm as a function of temperature. The resulting curves were normalized obtaining a plot of the folded fraction, *α*, versus temperature. The melting temperature (*T*_m_) was then deduced as the temperature corresponding to *α* equal to 0.5.
